# Caring task-involving climate leads to greater improvements in free-throw shooting biomechanics and motivational responses

**DOI:** 10.3389/fpsyg.2025.1667429

**Published:** 2025-10-15

**Authors:** Candace M. Hogue, Dimitrije Cabarkapa, Mary D. Fry, Andrew C. Fry, Troy O. Wineinger, Jacob M. Chamberlin

**Affiliations:** ^1^UMN Sport, Exercise & Performance Psychology Lab, School of Kinesiology, University of Minnesota, Minneapolis, MN, United States; ^2^Jayhawk Athletic Performance Laboratory - Wu Tsai Human Performance Alliance, Department of Health, Sport and Exercise Sciences, University of Kansas, Lawrence, KS, United States; ^3^KU Sport & Exercise Psychology Lab, Department of Educational Psychology, University of Kansas, Lawrence, KS, United States; ^4^School of Teaching, Learning, and Curriculum Studies, Kent State University, Kent, OH, United States

**Keywords:** skill development, achievement goal perspective theory, motivational climate, coaching, achievement goal theory

## Abstract

**Introduction:**

There is a need for experimental and cross-disciplinary research in sport psychology, particularly studies that incorporate objective performance assessments into motivational climate research.

**Methods:**

This investigation examined biomechanical changes in athletes’ free-throw shooting form in response to the motivational climate during a basketball clinic, as well as affect, demand and resource appraisals, and motivational outcomes. Thirty-nine male basketball players were assigned to a free-throw clinic with either a caring, task-involving (CTI) climate, where high effort and improvement are valued and recognized and mistakes are part of learning or an ego-involving (EI) climate, where winning is prioritized, athletes are punished for mistakes, and star players are favored. Participants completed pre- and post-clinic surveys. Video analysis allowed for the assessment of free-throw kinematics (e.g., knee flexion) pre- and post-clinic.

**Results:**

No baseline group differences were found. At post-assessment, the CTI group’s shooting kinematics more closely resembled those of proficient shooters compared to the EI group. Individuals in the EI climate perceived the clinic as more demanding and reported a significant increase in negative affect. In contrast, CTI participants reported significantly greater positive affect, effort, and interest and excitement to continue practicing.

**Discussion:**

Findings suggest creating a CTI climate can enhance motivation and facilitate player development.

## 1 Introduction

Sustaining motivation to engage in physical activity across the lifespan is one of the most pressing issues of our day, due to the numerous psychological and health benefits that are associated with physical activity (e.g., greater well-being, muscle strength, and immune functioning; Leblanc et al., 2015; Li et al., 2022; Marquez et al., 2020). Central to promoting continued sport and physical activity participation are helping individuals develop the desire to continue to enhance their skills and the ability to move skillfully, display high effort, and experience positive affect (Duda and Balaguer, 2007). One effective strategy in achieving these aims is creating positive and supportive motivational sport environments that bring out the best in each individual (Duda and Balaguer, 2007). Previously published research (Fontana et al., 2022; Gerabinis et al., 2018; Hogue, 2024, 2025; Solmon, 1996) suggests that when athletes experience an environment that primarily rewards performance outcomes and punishes athletes for making mistakes or losing, they may be less likely to maximize their skill development and sport experience. However, there are relatively few studies that incorporate more objective measures of performance (e.g., Frederick and Fry, 2017; Solmon, 1996) and skill development (e.g., Johnson et al., 2019) and more research is warranted on this topic. As such, the purpose of this experimental investigation was to examine whether the coach-driven motivational climate impacts objective measures of skill development, as well as motivation-related responses in athletes.

Nicholls (1989) described why a task-involving (TI) climate is the gold standard for environments created in sport and educational settings. His goal perspective theory has been readily employed in the physical domain and embraced by numerous researchers in the sport and exercise psychology field. Additionally, Nicholls (1992) and other achievement goal perspective researchers (Duda, 2001; Newton et al., 2010; Roberts, 2012) maintain that coaches create a TI climate when they help athletes, above all else, (a) feel successful when they give their best effort and display mastery over time; (b) adopt the view that mistakes are part of the learning process; (c) understand that every athlete plays an important role on the team; and (d) interact cooperatively with each member of the team. Newton et al. (2007) added an additional psychosocial feature to the TI climate that was based on Noddings (2003, 2005) caring framework. Specifically, Newton et al. (2007, p. 70) defined a caring climate as one that is “interpersonally inviting, safe, supportive, and able to provide the experience of being valued and respected.” These researchers suggested that a combined caring and task-involving (CTI) climate would enhance athletes’ likelihood of displaying optimal affective, cognitive, and behavioral responses in sport, as the benefits of developing supportive interpersonal relationships among participants, and participants and coaches, in a caring climate nicely compliment a TI climate where the focus is on skill development and utilizing a cooperative approach toward achievement. For instance, in an investigation with Division I athletes, caring climates were positively associated with greater coachability, while TI climates were positively linked to goal setting and achievement motivation (Fry et al., 2021). This example highlights the complementary but unique nature of both caring and TI climates–being receptive to feedback and implementing cues from coaches (e.g., coachability) would logically contribute to greater objective improvements in performance in environments where coaches provide individualized feedback to athletes, treat mistakes as part of the learning process, and reward athletes for trying hard (i.e., a TI climate).

In contrast to the CTI climate, Nicholls (1989, 1992) described the ego-involving (EI) climate as one that is perhaps more prominently observed in sport and educational settings, yet less effective in facilitating optimal achievement experiences. In an EI climate, coaches (a) have a disproportionate focus on winning and athletes’ normative performances; (b) more often respond to athletes’ mistakes with punishment; (c) give most of their attention to a few “stars” on the team; and (d) foster rivalry among teammates. Consistent support has emerged over the last 15 years that demonstrates how a CTI climate is linked to athletes reporting more effective learning and coping strategies, and in some cases better sport technique development and performance, in comparison to more problematic responses observed in an EI climate (For reviews see Fry and Moore, 2019; Gano-Overway and Fry, 2024). For example, when athletes and college students enrolled in physical activity courses perceived a TI climate, they were more likely to report using adaptive learning strategies such as goal setting, attentional focus, utilizing coach feedback, and practicing alone outside of the team practice (Boyce et al., 2009; Gano-Overway and Ewing, 2004). Likewise, research with elite collegiate athletes have linked caring and TI climates to greater ability to peak under pressure, concentrate, experience freedom from worry, cope with adversity, and use approach coping strategies (Fry et al., 2021; Kim et al., 2011).

It follows that if athletes are motivated to try hard (e.g., recognized for high effort), work collaboratively to learn and develop their skills, are supported and encouraged by their team, and treat mistakes as opportunities to learn (i.e., are immersed in a CTI climate), they would adopt more effective learning strategies and develop their skills at a more rapid pace than if they are punished for mistakes, are made to feel embarrassed when outperformed by a teammate, and are given less feedback compared to the higher performing athletes (i.e., are immersed in an EI climate). Likewise, Nicholls emphasized the importance of positive interpersonal interactions and social support for optimizing the motivation and experience of all participants in achievement contexts (Nicholls and Hazzard, 1993). Newton et al. (2007) continued his work on the importance of creating a caring climate in sport and described the value of athletes feeling accepted for who they are, cared for, and respected by others. The research has shown that caring climates lead to better relationships (Fry and Gano-Overway, 2010) and athletes caring about their health and their teammates’ health (Brown et al., 2019), while TI climates consistently lead to greater effort (For a review see Fry and Moore, 2019). Annerstedt and Lindgren (2014) offer an example of a high performing coach that helps illustrate the complimentary nature of caring and TI climates in sport. In their case analysis with a national level Swedish coach, the coach explains that knowing his athletes on an individual level is not only important to build the relationship but also to understand how to support them in reaching their performance potential.

There is some, albeit limited, research that incorporates performance measures suggesting that athletes’ immersion in a CTI climate could enhance their effective use of learning strategies and mental skills, along with their sport skill development (e.g., technique). In an early investigation, Theeboom et al. (1995) found that youth in a summer sport camp who experienced a mastery (TI) climate received higher ratings of accuracy and proficiency in their performance of sport skills, relative to a comparison group with more of an EI focus. A later study with elite female handball athletes found TI climates predicted greater perceived performance improvement (e.g., tactical, physical) while EI climates were found to be unrelated to athlete perceptions of performance (Balaguer et al., 2002). An investigation conducted by Moles et al. (2017) found male soccer players performed a shooting task more effectively when placed in a TI climate compared to those placed in an EI climate, and in a similar experimental investigation Reinboth and Duda (2016) found college students’ objective effort was significantly greater when placed in a TI climate compared to an EI climate, regardless whether they were told they were on track to win or lose their race. Finally, in a more recent investigation with collegiate basketball players, highly CTI climates were associated with more assists and fewer personal fouls, while EI climates were negatively related to assists (Frederick and Fry, 2017). Interestingly, no significant positive associations were observed for skill development in these previous investigations when athletes perceived an EI climate. Although there is great value in these findings, this remains an under-investigated topic.

In addition to sport skill development, another important benefit of athletes experiencing a CTI climate is their ability to manage performance stress, compared to athletes immersed in an EI climate. When athletes appraise performance contexts as challenges rather than threats, their physiological and psychological responses have been shown to help facilitate their learning and performance. For instance, when evaluations of resources outweigh evaluations of demands this reflects a challenge appraisal, which has been linked to more adaptive cardiovascular reactivity, as well as athletic performance (Blascovich et al., 2004; Doron and Martinent, 2021; Turner et al., 2021). Challenge appraisals refer to evaluative, goal-oriented performance contexts where one is invested or cares about their performance and perceives the situation as a positive challenge, whereas resource evaluations refer to the belief that one has, or might have, the ability to perform well and they expect to perform well. In contrast, demand evaluations are defined as perceptions that an evaluative, goal-oriented context is distressing, threatening, and demanding, and where a strong performance is uncertain. Mendes et al. (2007) utilize a threat index to reflect one’s evaluation as to whether they feel they have the resources to meet or manage the demands in such performance contexts. When evaluations of demands outweigh resource evaluations, this reflects a threat appraisal, which has been linked to less adaptive outcomes in sport, including performance outcomes (Blascovich et al., 2004).

One strategy that has been utilized in the sport psychology literature to understand the impact of the motivational climate is to conduct experimental research. For instance, a series of experimental investigations have been conducted by Hogue and colleagues in order to compare participant responses while learning a new skill (i.e., juggling) in either a CTI climate or EI climate. These investigations have revealed reports of greater positive affect (Hogue, 2024; Hogue et al., 2017, 2021), effort (Hogue et al., 2013, 2017), and enjoyment (Hogue et al., 2013, 2017) and lower negative affect (Hogue, 2024; Hogue et al., 2017, 2021) by those immersed in a CTI climate. Moreover, CTI climates were found to elicit a significant rise in positive affect, compared to pre-climate exposure, whereas EI climates were found to elicit a significant rise in negative affect (Hogue, 2019, 2020, 2024). Positive affect, as defined by Watson et al. (1988), includes feeling enthusiastic, active, and alert, whereas experiencing distress and other indicators of unpleasant engagement, such as feeling irritable, scared, and ashamed, are reflective of negative affect. Experiencing positive emotions while engaged in sport has been shown to have a favorable impact on athlete engagement, self-efficacy, and motivation to continue practicing and developing their skills (McCarthy, 2011). Therefore, an EI climate may serve to undermine athletes’ development of a strong work ethic and commitment to continued growth over time, whereas a CTI climate likely serves to facilitate these adaptive motivational responses.

The aforementioned experimental investigations built on Solmon’s (1996) early experimental work where she taught youth to juggle in either a TI or EI climate and observed their responses to learning this new skill in a physical education class among their peers. Juggling is an ideal physical activity to incorporate in climate research because when individuals who lack the skill are recruited, a level playing field is created, where participants begin at a novice level. However, juggling does not create the same level of intensity that may be observed in sport-specific interventions, and Hogue et al. (2017) have called for climate interventionists to include more mainstream sport skills. It may be, for instance, that in sport settings athletes would feel more confident in their ability to perform well and would expect to perform better (i.e., have higher resource evaluations) than when learning a new skill. Although, it is plausible, given the normative comparisons, punishment after mistakes, and strong emphasis on winning and outperforming others in EI climates that athletes’ demand evaluations, including distress and performance uncertainly, would be high, even in a familiar sport context. For instance, EI climates have been shown to elicit a multifaceted stress response in athletes (Hogue, 2020), including Division-I student-athletes (Hogue, 2025a), as well as college students (Breske et al., 2017; Hogue, 2019, 2024; Hogue et al., 2013, 2021) and middle school students (Hogue et al., 2017). Further research is needed to understand the impact of the motivational climate on athletes who are working on further developing skills for a sport they have played and have developed sport skills in, as these relationships have not been experimentally investigated in a sport context.

Within the current climate literature in sport, the assessment of objective skill technique is limited. Clearly, developing effective sport skills is central to sport and often necessary for athletes to remain engaged and motivated to continue to be involved over time (Balish et al., 2014). One reason researchers in sport and exercise psychology have not examined sport technique and objective performance outcomes more extensively in relationship to the sport climate is because of the necessity of conducting cross-disciplinary research (Weiss and Gill, 2005). Weiss (2020) highlights the value of conducting cross-disciplinary work to further our understanding of differences in sport performance and physical activity engagement by capturing domain specific measures of motor competence, in addition to perceived motor competence. Likewise, in her review of coach-athlete relationship research, Jowett (2025) has also called for more cross-disciplinary research in order to advance knowledge and understanding in sport.

We contend that while there is great value in understanding subjective perceptions of effort and ability, to really test achievement goal theory both types of assessments (objective and subjective) are necessary. In a retrospective review of their goal setting theory, Locke and Latham (2019) made a similar argument, indicating that it is important to show generalizability of theories by replicating work with different types of methodologies, including variations in measures used and populations assessed. Likewise, in their systematic review of the intrapersonal correlates of motivational climate perceptions in sport and physical activity, Harwood et al. (2015) noted that objective measures are rare (but important) in climate research and recognized that most research assessing affective states and climate perceptions do not attempt to establish direction of causality. Each of these relationships and their directionality are important for effectively testing theory and extending our goal perspective theory knowledge base. Harwood et al. (2015, p. 19) also argued, “in order for research findings to produce meaningful recommendations about the objective determinants (and outcomes) of [motivational] climate perceptions, it may be necessary to introduce some more ambitious and innovative measurement techniques.” We agree, and note that providing objective, biomechanical evidence in support of the creation of a CTI approach toward skill development with athletes will also help address the misconception that “tough love” and a win-at-all costs mentality facilitates performance.

Therefore, an important area of inquiry to advance the sport climate research is to consider how distinct motivational climates impact athletes’ objective sport skill development, in addition to various indicators of motivation. As such, the purpose of this investigation was to examine whether the motivational climate (i.e., CTI vs. EI) during a basketball free-throw shooting clinic impacts athletes’ shooting form, perceived ability to manage performance stress, effort and affect during the clinic, as well as motivation to continue developing their skills. It was hypothesized that post-clinic, those randomly assigned to the CTI climate would demonstrate greater objective skill improvement (i.e., more proficient shooting technique), report more adaptive responses to performance stress (e.g., lower demand evaluations), and greater motivational responses (i.e., greater positive affect, effort, and interest in continuing to practice their skills), compared to those in an EI climate.

## 2 Materials and methods

This research was part of a larger investigation (Hogue et al., 2025). Relevant details for this portion of the investigation are included below.

### 2.1 Participants

Thirty-nine males [*M_age_* = 20.2 ± 1.8 years; height = 180.67 cm (71.13 in.), *SD* = 25.78 cm; body mass = 76.85 kg (169.43 lbs.), *SD* = 12.08 kg] with at least 4 years basketball experience who were attending college at a large Midwest University volunteered to participate in the present investigation. In order to meet inclusion criteria, participants must have played basketball at the high school level or below and must have played within the last 6 months (e.g., recreation basketball league). Approximately half of the participants participated in high school basketball and all of the participants were involved in basketball at the youth sport level. Potential participants were recruited via fliers posted around campus and through word of mouth. Participants were primarily Caucasian (82%) and Hispanic/Latino (11%) and were randomly assigned to a free-throw shooting clinic with either a CTI climate (*n* = 20) or an EI climate (*n* = 19). Each participant was paid $25 for taking part.

### 2.2 Procedure

The study was approved by the Institutional Review Boards at each of the authors’ universities. Data collection took place on weekday afternoons with three waves of 6–7 participants taken through the free throw shooting clinic each day (i.e., CTI Day 1; EI Day 2). Each group had four instructors and two confederates (i.e., planted participants) who took part in a minimum of 8 h of training in order to help ensure the creation of each climate. Instructor training consisted of weekly meetings, for a total of 9.5 h, where graduate and undergraduate research assistants, along with faculty, discussed climate research, practiced developing and carrying out the free throw shooting clinic, and received feedback on their delivery during mock free throw clinics. Instructors and confederates were given feedback from students and faculty familiar with the defining features of each respective climate.

**Figure 1:**
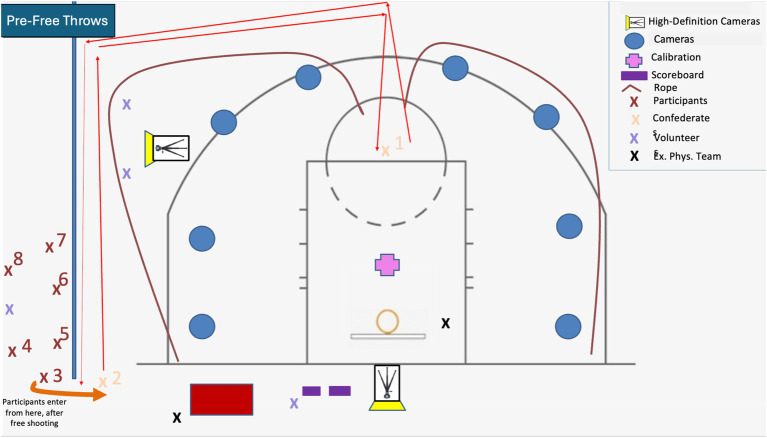
Kinovea V0.8.15 cameras and high-definition camera placement and participant movement for kinematic assessment.

The study was introduced to participants when they arrived, and consent was requested. Participants completed the pre-clinic surveys in order to assess baseline affect just before the start of the free-throw shooting clinic. Then, participants were taken to a gym where they warmed up for approximately 5 min. One by one, participants shot ten free throws while the mechanics of their free-throw shooting motions (sagittal and frontal planes of motion) were assessed via video analysis software (Kinovea V0.8.15, Bordeaux France). See Figure 1 for graphical representation and the biomechanical assessment section below. Once each person completed the baseline set of free-throw shots, they took part in the 20-min free-throw shooting clinic with either a CTI climate or EI climate [See Experimental Manipulation section below or Hogue et al. (2025) for a detailed explanation]. Post-biomechanical skill assessments were conducted for each participant while they were still immersed in their respective climate (25 min) and were in the presence of the other participants in their group, as well as the instructors, confederates, and researchers. Upon completion of the biomechanical assessment (i.e., video analysis), the participants returned to the classroom where they completed post-session questionnaires and were debriefed as to the true nature of the investigation. Specifically, participants were told that the purpose of the investigation was to compare how athletes respond to the motivational climate fostered by coaches. A brief explanation of a CTI climate versus an EI climate was included and the climate literature was summarized.

### 2.3 Experimental manipulation

Each clinic began with an icebreaker to reinforce the climate features, which was followed by four instructional free-throw shooting activities. A total of five free throw shooting cues were taught in each climate, as detailed by Cabarkapa et al. (2021a) and Cabarkapa et al. (2021b). Cues one and two entailed squaring feet and shoulders up to the basket and having a deep, comfortable knee bend, which were taught and practiced during Activity 1. Cues three and four entailed tucking in the elbow and releasing the ball at the peak of one’s shot, which were taught and practiced during Activity 2. The fifth cue entailed aiming at the back half of the rim, which was taught and practiced during Activity 3. Activity 4 was a final competition that differed by climate and is explained below, where participants practiced implementing all of the cues.

#### 2.3.1 CTI climate condition

Throughout the CTI climate clinic, instructors and confederates worked to create a welcoming environment for the participants, recognized their improved form, encouraged participants to support one another, and praised participants for trying hard. The instructors also provided individualized feedback and emphasized that mistakes are part of the learning process. The icebreaker in the CTI climate began with participants sharing a favorite sports memory or a sports memory that brings them joy. Each of the four activities that followed began with an explanation and demonstration of the cues, followed by each participant shooting a total of six free throws (three at a time) while the other participants observed and/or practiced as they rotated around the basket and were immersed in a CTI climate. For the final activity, Activity 4, participants were randomly split into two teams, each with two instructors and one confederate, for a final competition. The purpose was to practice their free-throw shooting skills and to work to improve the number of shots they made individually and as a team while shooting a total of 6 free throws (three at a time). This was repeated for a final competition; however, each participant shot 10 free throws at a time. Each individual participant’s score and their team’s score were tallied on a portable scoreboard that was in view of all participants and instructors. The instructors and confederates did not draw attention to the score.

#### 2.3.2 EI climate condition

Throughout the EI climate clinic, the instructors treated the confederates as the star athletes, pitted participants against one another and compared their skills to the confederates’ skills, emphasized the importance of winning and outperforming others, and punished participants for making mistakes (e.g., moved them closer to the basket). The EI climate began with an icebreaker called “glory days” (Hogue et al., 2013) where participants were asked to share their greatest sport accomplishment with the group, while the instructors and confederates created an EI climate (e.g., acted impressed by records or high scores). Each of the four activities that followed began with an explanation and demonstration of the cues, followed by each participant shooting a total of six free throws (three at a time) while the other participants observed and/or practiced as they rotated around the basket and were immersed in an EI climate. In the EI climate, the final activity, Activity 4, began with the instructors picking teams, one participant at a time, and then each participant shot a total of six free throws (three at a time) while immersed in an EI climate. Prior to the final competition, the purpose of the competition was explained as “to see who had the best shooting team.” Each individual participant’s score and their team’s score were tallied on a portable scoreboard that was presented to all participants and instructors. The instructors and confederates drew attention to the score with regularity. The participants then shot 10 free-throws at a time for the competition.

### 2.4 Biomechanical assessment

To evaluate kinematic changes in free-throw shooting technique, video analysis was conducted using Kinovea software (version 0.8.15). The data was analyzed manually by experts who have examined more than 10,000 shots with an error rate of < 2%. Two high-definition cameras were positioned 10 meters from the free-throw line to capture the shooting motion from both the sagittal and frontal planes. Five biomechanical variables were analyzed for each of the ten free-throw attempts during both the pre- and post-clinic assessments: (1) ankle angle, (2) knee flexion, (3) elbow height, (4) release height, and (5) peak ball trajectory (i.e., “basketball peak”). Kinematic changes were assessed by calculating the percent change from pre- to post-clinic measures and by comparing participants’ post-clinic mechanics to those of proficient shooters, as defined in prior research (Cabarkapa et al., 2021a; Cabarkapa et al., 2021b).

### 2.5 Manipulation check

#### 2.5.1 Motivational climate perceptions

The Perceived Motivational Climate in Sport Questionnaire (PMCSQ; Seifriz et al., 1992) and Caring Climate Scale (CCS; Newton et al., 2007) were used to assess participant perceptions of a TI climate, EI climate, and caring climate during the free throw clinic in order to ensure the intended climate was observed by the participants during each respective clinic. Both the PMCSQ and CCS use a Likert-style scale ranging from 1 (*strongly disagree*) to 5 (*strongly agree*), and scores were averaged for a final composite score. The stem for each scale was altered to read, *“*During the basketball clinic….” The PMCSQ has 21-items and measures the extent to which the climate was perceived to be a TI climate and an EI climate during the free throw clinic. A TI climate example item is “…each participant’s improvement was important.,” while an example EI item example is “…participants were afraid to make mistakes*.”* The CCS has 13-items and measures the extent to which the environment is safe, caring, and participants are treated with respect. An example item is, “…the instructor was kind to the participants.” Previous research has found the PMCSQ and CCS display adequate psychometric properties (Newton et al., 2007; Seifriz et al., 1992).

### 2.6 Potential confounding factor

#### 2.6.1 Basketball experience

Participants were asked to share the highest level (e.g., high school, AAU) of basketball they played, in order to help assess whether random assignment was successful in balancing the potential skill level of the groups.

### 2.7 Psychological outcomes

#### 2.7.1 Pre- and post-questionnaire

##### 2.7.1.1 Affect

The Positive Affect and Negative Affect Schedule (PANAS; Watson et al., 1988) was used to assess positive and negative affect immediately prior to and during the free throw clinic. Positive affect includes feelings of interest and excitement, for example, while negative affect includes feelings of hostility and irritability. Each subscale included 10-items with a Likert scale that ranged from 1 (*not at all*) to 5 (*extremely*). Items for each subscale were summed for a composite score. The stems “I feel…” (pre) and “During the basketball clinic, I felt…” (post) were used. The PANAS has strong psychometric properties (Watson et al., 1988) and has been used in other similar experimental investigations (e.g., Hogue, 2024, Cronbach’s alpha = 0.91 for positive affect and 0.87 for negative affect).

#### 2.7.2 Post-only questionnaire

##### 2.7.2.1 Challenge and threat appraisals

Participants’ demand and resource evaluations during the free-throw clinic were assessed using Mendes et al. (2007), the challenge and threat measure. Demand evaluations reflect feelings of stress, expectation, and uncertainty when performing in an achievement setting, while resource evaluations reflect perceived ability to cope with potential stressors. Items were adjusted to better represent the free-throw clinic. This scale included two subscales made up of six demand and five resource evaluation questions. A Likert scale that ranged from 1 (*strongly disagree*) to 7 (*strongly agree*) was used. Example items were “The free throw clinic was demanding” (demand) and “I expected to perform well during this free throw clinic” (resource). A threat or challenge appraisal was calculated by using the average demand/average resource ratio. A ratio of *>*1 reflects a threat appraisal, while a *<* 1 ratio reflects a challenge appraisal (Mendes et al., 2001). This measure has displayed acceptable psychometric properties (Mendes et al., 2007) and has been used in other similar experimental investigations with athletes (e.g., Hogue, 2025a; Cronbach’s alpha = 0.86 for threat appraisals and 0.70 for challenge appraisals).

#### 2.7.3 Motivational responses

##### 2.7.3.1 Effort

The five item effort subscale of the Intrinsic Motivation Inventory (McAuley et al., 1989) was used as a subjective measure of effort during the basketball clinic. Responses ranged from 1 (*strongly disagree*) to 7 (*strongly agree*) and an average score was calculated for the scale. An example effort item was “I put a lot of effort into this basketball clinic.” This measure has displayed acceptable psychometric properties (McAuley et al., 1989) and has been used in other similar experimental investigations (e.g., Hogue et al., 2017; Cronbach’s alpha = 0.88).

##### 2.7.3.2 Interest and excitement to continue practicing cues

Two items were developed in order to assess participants’ interest and excitement to continue practicing the cues they were taught during the free throw shooting clinic. These individual items were assessed using a Likert style scale that ranged from 1 (*not at all*) to 7 (*very much so*).

### 2.8 Statistical analysis

There was approximately 1% missing data for the psychological responses. As a result, the average of each respective scale was used to replace the missing psychological responses. There was no missing data for the biomechanical assessments of free-throw shooting form. Perceptions of the motivational climate during the clinic were verified through a manipulation check. In order to determine whether the intended motivational climate (i.e., CTI vs. EI) was perceived by participants in each respective group, paired samples *t*-tests were run within each climate group. To test the hypotheses, participants were separated into either the CTI climate or EI climate group, which was treated as the between-subjects variable. In order to assess differences in free-throw kinematic parameters by motivational climate, a MANOVA was run that included post-free throw clinic percent change from baseline (i.e., pre-free-throw clinic) for ankle angle, knee flection, elbow height, release height, and basketball peak as the within-subjects variables. Affect was assessed using 2 (Climate: CTI climate vs. EI climate) × 2 (Time: pre- vs. post-free-throw clinic) mixed-design, repeated-measures MANOVA. Climate was treated as the between-subjects variable, and time was treated as the within-subjects variable. Post-only variables were assessed using MANOVAs or an ANOVA (effort) and were grouped as explained in the results section below. Cohen’s *d* was calculated in order to assess the magnitude of group differences for the psychological variables, which are interpreted as large for 0.80 or greater, moderate for 0.50 to 0.79, and small for 0.20 to 0.49 (Cohen, 1988).

## 3 Results

See Table 1 for kinematic characteristics of free throw shooting form pre- and post-clinic by motivational climate compared to scores of proficient shooters. See Table 2 for means and Cohen’s *d* for psychological outcomes. Table 3 includes correlations between climate perceptions and psychological measures including positive and negative affect, demand and resource evaluations, threat/challenge appraisals, effort, and interest and excitement to continue practicing, as well as Cronbach’s alphas for each respective scale.

**Table 1:** Means and standard deviations (sd) of percent change in kinematic characteristics of free throw shooting by motivational climate.

Parameter (degrees)	Proficient Shooters	CTI group			EI group		
		Percent change	Pre	Post	Percent change	Pre	Post
Ankle angle (degrees)	52.6 (3.9)	−0.57 (7.08)*	52.73 (7.43) degrees	52.69 (5.48) degrees	−4.45 (5.25)*	59.13 (7.29) degrees	56.30 (5.85) degrees
Knee flexion angle (degrees)	101.1 (8.1)	−1.02 (5.31)	114.47 (12.46) degrees	112.88 (9.73) degrees	−3.68 (3.99)	122.79 (13.72) degrees	117.91 (10.27) degrees
Elbow height (ratio)	*–*	0.97 (5.22)	113.12 (20.35)	114.31 (21.87)	−1.56 (3.04)	121.45 (22.54)	119.49 (22.18)
Release height (ratio)	*–*	5.16 (7.21)*	228.51 (13.31)	233.67 (13.95)	−0.92 (5.40)*	232.50 (17.5)	231.58 (16.20)
Basketball peak (ratio)	–	0.95 (3.74)*	145.63 (21.35)	146.99 (21.99)	−1.90 (4.65)*	146.32 (31.48)	143.77 (32.28)

Lower positioning in elbow height and higher positioning in release height and basketball peak are considered advantageous. CTI climate refers to the caring, task-involving climate group. EI climate refers to the ego-involving climate group. * Represents significant differences between groups at *p* < 0.05.

**Table 2:** Means (*SD*), Cohen’s *d*, and scales for pre- and post- and post-only psychological measures by motivational climate.

Variable	Cohen’s *d*	CTI group	EIgroup	Scale
Pre-to-post variables affect				
Pre-positive affect		29.85 (7.06)^τ^	30.26 (5.63)	[10–50]
Post-positive affect	1.01	35.70 (7.31)^τ a^	28.26 (7.37)^a^	[10–50]
Pre-negative affect		13.20 (3.40)	13.63 (3.47)^x^	[10–50]
Post-negative affect		14.50 (4.07)	15.94 (4.64)^x^	[10–50]
Post-only variables Challenge and threat appraisals				
Demand evaluations	0.78	2.58 (0.85)^b^	3.39 (1.20)^b^	[1–7]
Resource evaluations		5.19 (1.02)	5.14 (1.03)	[1–7]
*Threat ratio (demands/resources)*	0.61	0.53 (0.22)^b^ *Challenge Appraisal*	0.68 (0.27)^b^ *Challenge Appraisal*	< 1 *Challenge Appraisal* > 1 *Threat Appraisal*
Motivational responses				
Effort	0.88	5.35 (1.27)^a^	4.34 (1.01) ^a^	[1–7]
Interest in cont. to practice cues	1.26	5.85 (1.23)^a^	3.89 (1.82)^a^	[1–7]
Excitement to cont. practicing cue	1.34	5.95 (1.05)^a^	3.95 (1.84)^a^	[1–7]

CTI Climate refers to the caring, task-involving climate group. EI Climate refers to the ego-involving climate group. Same subscripts note significant differences between groups (i.e., CTI Climate vs. EI Climate) at *p* < 0.01 for ^a^ and *p* < 0.05 for ^b^ or within groups from pre- to post-clinic at *p* < 0.01 for ^τ^ and *p* < 0.05 for ^x^.

**Table 3:** Correlation table among motivational climates and post-free throw clinic psychological measures and Cronbach’s alphas.

Variables	1	2	3	4	5	6	7	8	9	10	11
1. Caring Climate	1										
2. Task Climate	0.68**	1									
3. Ego Climate	−0.93**	−0.55**	1								
4. Demand Appraisals	−0.46**	−0.07	0.57**	1							
5. Resource Appraisals	0.12	0.12	0.04	0.04	1						
6. Threat Index	−0.47**	−0.15	0.50**	0.86**	−0.46**	1					
7. Effort	0.48**	0.41**	0.12	−0.05	0.35*	−0.20	1				
8. Interest in Continuing	0.67**	0.59**	0.15	−0.09	0.12	−0.11	0.59**	1			
9. Excitement in Continuing	0.69**	0.61**	0.09	−0.15	0.14	−0.17	0.55**	0.97**	1		
10. Positive Affect	0.62**	0.60**	−0.51**	−0.15	0.32	−0.26	0.47**	0.62**	0.61**	1	
11. Negative Affect	−0.21	−0.05	0.22	−0.11	−0.15	0.32	−0.26	0.47**	0.62**	0.61**	1
Cronbach’s alpha	0.97	0.71	0.97	0.90	0.76	0.79	0.62	–	0.84	–	–

** Correlation at *p* < 0.01, *p < 0.05.

### 3.1 Manipulation check

#### 3.1.1 Motivational climate perceptions

Participants in the CTI climate group reported perceiving a significantly more caring [*M* = 4.67, *SD* = 0.34; *t*(1, 19) = 21.38, *p* < 0.001] and TI climate [*M* = 4.15, *SD* = 0.51; *t*(1, 19) = 20.00, *p* < 0.001], compared to an EI climate (*M* = 1.59, *SD* = 0.40). Likewise, participants in the EI climate perceived a significantly more EI climate (*M* = 3.81, *SD* = 0.47), compared to caring [*M* = 2.78, *SD* = 0.69; *t*(1, 18) = 4.01, *p* < 0.001] and TI climate [*M* = 3.43, *SD* = 0.50; *t*(1, 18) = 2.36, *p* = 0.03]. These findings indicate the instructors and confederates were successful in creating each respective motivational climate.

### 3.2 Potential confounding factor

#### 3.2.1 Basketball experience

There were no significant differences between the CTI climate and EI climate groups’ previous basketball experience, *F*(1, 37) = 3.69, *p* = 0.06.

### 3.3 Biomechanical assessment–free-throw shooting kinematics

Results revealed a significant main effect for Climate, *F*(5, 31) = 3.16, *p* = 0.020, η2 = 0.34. Follow-up analyses revealed a significant difference between the CTI climate and EI climate groups for percent change in ankle angle, *F*(1, 35) = 5.83, *p* = 0.021, η2 = 0.14, release height, *F*(1, 35) = 7.55, *p* = 0.009, η2 = 0.18, and basketball peak, *F*(1, 35) = 4.29, *p* = 0.046, η2 = 0.11, with participants in the CTI climate group responding more favorably. The ankle angle and knee flexion of the CTI climate group moved closer to proficient, and both the release height and basketball peak increased, indicating improvements in form. The elbow height for the EI climate group decreased, and the knee flection of the EI climate group moved towards those of proficient shooters, which is advantageous. However, the EI climate group release height and basketball peak both decreased, which is not considered an improvement on their form. The differences between groups were not significant for percent change in knee flexion, *F*(1, 35) = 2.88, *p* = 0.10 or elbow height, *F*(1, 35) = 3.10, *p* = 0.09.

### 3.4 Pre-post-psychological outcomes

#### 3.4.1 Affect

Results revealed a non-significant main effect for Climate, *F*(3, 35) = 0.37, *p* = 0.77, and a non-significant main effect for Time (pre vs. post), *F*(3, 35) = 1.76, *p* = 0.17, and a significant Time x Climate interaction, *F*(3, 35) = 6.89, *p* < 0.001, η2 = 0.37. There were no significant baseline differences in positive affect, *F*(1, 37) = 0.04, *p* = 0.84, or negative affect, *F*(1, 37) = 0.15, *p* = 0.70. There were, however, significant differences in positive affect during the free throw clinic, *F*(1, 37) = 10.00, *p* = 0.003, η2 = 0.21, with the CTI climate group reporting more positive affect. There were no significant differences between groups in negative affect during the clinic *F*(1, 37) = 1.08, *p* = 0.31. Follow-up analyses also revealed a significant increase in positive affect for participants in the CTI climate group, *t*(1, 19) = 3.64, *p* = 0.002, and a rise in negative affect for participants in the EI climate group, *t*(1, 18) = 2.68, *p* = 0.020, during the free-throw clinic, compared to just prior to the start of the clinic. There were no significant differences in pre- to post-negative affect for the CTI climate group, *t*(1, 19) = 1.53, or pre- to post-positive affect for the EI climate group, *t*(1, 18) = 1.34, *p* = 0.20.

### 3.5 Post only psychological outcomes

#### 3.5.1 Challenge and threat appraisals

The main effect for Climate was non-significant for demand and resource evaluations, *F*(2, 36) = 2.97, *p* = 0.06. Follow-up analyses revealed group differences for demand evaluations, *F*(1, 37) = 5.83, *p* = 0.021, η2 = 0.14, but not resource evaluations, *F*(1, 37) = 0.14, *p* = 0.71, with the EI climate group reporting significantly greater demands during the clinic. The threat index (demand/resource evaluations; Mendes et al., 2001) was also significantly greater for the EI climate group, *F*(1, 37) = 5.94, *p* = 0.020, η2 = 0.14, although both groups felt they had the resources needed to meet the demands during the free throw clinic (i.e., their threat index was < 1), indicating a challenge appraisal.

#### 3.5.2 Effort

There was a main effect for Climate, *F*(1, 37) = 7.44, *p* = 0.010, η2 = 0.17 with the CTI climate group reporting putting forth greater effort during the clinic.

#### 3.5.3 Interest and excitement to continue practicing cues

There was a significant main effect for Climate, *F*(2, 36) = 8.50, *p* < 0.001, η2 = 0.32 for interest and excitement to continue practicing. The CTI climate participants reported much greater interest in (*M_EI_* = 3.89, *M_CTI_* = 5.85), *F*(1, 38) = 15.60, *p* < 0.001, η2 = 0.30 and excitement to (*M_EI_* = 3.95, *M_CTI_* = 5.95), *F*(1, 38) = 17.65, *p* < 0.001, η2 = 0.32 continue practicing the cues they were taught during the free throw clinic.

## 4 Discussion

The purpose of this experimental investigation was to examine whether the motivational climate during a sport clinic might impact objective measures of performance (i.e., improvement) in former athletes familiar with the sport, as well as psychological factors likely to facilitate skill development over time. Specifically, the biomechanics of free-throw shooting form and affect were assessed prior to and following a free throw shooting clinic with either a CTI climate or an EI climate. Participants’ (i.e., former basketball players) ability to manage performance stress during the clinic was also assessed, along with their self-reported effort and interest and excitement in continuing to practice the skills they were taught during the free-throw clinic. Considerable support was observed for the multiple hypotheses that a CTI climate would facilitate skill development and elicit more adaptive motivational and stress responses, compared to an EI climate. The CTI climate group displayed better shooting form post-clinic, compared to the EI climate group, and reported greater positive affect, effort, and interest and excitement to continue developing their free-throw skills indicating that over time, CTI climates will help maximize the development and performance of athletes. In contrast, participants in the EI climate group reported an increase in negative affect and greater demand evaluations compared to the CTI climate group indicating that over time, EI climates will hinder development and may adversely impact performance.

The biomechanical analyses of free-throw shooting technique utilized in this study is a unique addition to the sport psychology motivational climate literature. It is rare to see cross-disciplinary research in the field (e.g., Wiese-Bjornstal and Weiss, 1992), though it is important for advancing our understanding of how to maximize athletes’ physical and psychological development. Our findings align with previous cross-disciplinary work examining the impact of the motivational climate on motor skill development and performance. Theeboom et al. (1995) objectively assessed motor skill development in youth by videotaping (recording) and coding specific movements (i.e., level of wushu skill). They found that youth participants’ skills were objectively more advanced when taught in a mastery (task-involving) climate, compared to a control group learning these same skills, and argued that assessing the efficacy of a youth sport program on developing sport skills would have limited practical value without the objective assessment (p. 300). Reinboth and Duda (2016) compared the objective effort of college students cycling in a TI climate to those in an EI climate and found the TI climate led to greater meters biked compared to EI climates. Importantly, differences in subjective effort only approached significance (*p* = 0.10; with the TI climate group reporting greater exertion), highlighting how critical it is to have multiple assessments that incorporate both objective and subjective measures.

Partnering with professionals who have biomechanical expertise is key for understanding how the motivational climate created by coaches may influence sport skill development. The current study utilized video biomechanical analyses to consider the proficiency of the athletes’ free-throw shooting technique. The greater gains in the CTI climate group in three of the five parameters after a brief, 45-min free-throw shooting clinic and competition highlight a distinct difference in athlete development when they are exposed to EI versus CTI approaches to coaching. Participants in the EI climate group were given the same coaching cues as the CTI climate group and the same amount of time to develop their skills. If such differences can be captured after just 20 min of coaching, followed by a 25-min competition, it seems quite probable that teams with CTI climates will help maximize athlete skill development over time.

Overall, the biomechanical results favor the creation of a CTI climate for performance and skill development, with the CTI climate group displaying more proficient free-throw shooting technique after the clinic compared to the EI climate group. Specifically, the CTI climate group demonstrated ankle angle, release height, and basketball peak values that more closely resembled those of proficient basketball shooters compared to their peers in the EI climate group (Cabarkapa et al., 2023; Cabarkapa et al., 2021a; Cabarkapa et al., 2021b). The CTI climate group also showed improvements from pre- to post-clinic on four of the five parameters, including ankle angle, knee flection, release height, and peak basketball height. Only the elbow angle showed no significant changes. While the EI climate group displayed improvement with a decreased elbow bend and increased knee flection, they performed worse on their ball release height and basketball peak height. Considered together, the free-throw shooting form of the CTI climate group improved to a greater extent than the EI climate group. These results are noteworthy, particularly since the clinic was so brief, and there were no differences in free-throw shooting technique between the groups pre-clinic.

The psychological responses reported by participants in the CTI climate group add additional support for the contention that CTI climates will facilitate the development of all players, to a greater extent than EI climates. In the current investigation, the greater effort and positive affect during the free-throw clinic reported by participants in the CTI climate group suggest they had a positive experience and were motivated to develop their skills. Those in the EI climate group had a markedly different experience, reporting significantly lower positive affect and a significant rise in negative affect from baseline to post-clinic. Negative affect reflects to feeling upset, irritable, and distressed, whereas positive affect reflects to feeling attentive, inspired, and proud. These differential experiences suggest athletes who perceive a CTI climate on their teams will benefit more from each practice and are more likely to continue playing over time. The combination of high effort and motivation are key factors in optimizing athletes’ development and sustained participation.

Numerous previous studies have linked athletes’ perceptions of a CTI climate at all levels to their effort within and commitment to sport, interest in continuing their sport, and enjoyment (for a review see Fry and Moore, 2019). For instance, in research with youth athletes, Newton et al. (2010) linked perceptions of a TI climate to greater effort and enjoyment, while Curran et al. (2015) found positive associations between TI climates and various markers of engagement including confidence, vigor, dedication, and enthusiasm. Research also suggests more advanced (e.g., collegiate and Olympic athletes) are also motivated by CTI climates. In a recent study with collegiate athletes, Hogue (2025b) linked perceptions of a caring climate on NCAA Division I and III teams teams to greater sport enjoyment in athletes, and Pensgaard and Duda (2002) found evidence that even at the Olympic level athletes are responding favorably to more TI climates. Across studies, these important positive motivational outcomes are not typically observed within EI climates.

While this is the first experimental investigation to objectively examine how a CTI versus EI climate may differentially impact skill development, it would be valuable to look at longer term skill development and performance both in experimental settings and on sport teams. Since differences were observed in such a short period, one wonders how striking differences might be across a season, when athletes experience an environment where coaches recognize and value their effort and improvement and strive to build genuine relationships with their athletes. When the coach-athlete relationship is built around establishing caring interactions, this allows for the coach to understand an athlete and provide unique feedback that can lead to targeted improvement (Gano-Overway and Carson Sackett, 2021), in contrast to an EI climate where coaches are most concerned with performance outcomes, and there is little tolerance for mistakes. Furthermore, athletes experiencing an EI climate may be at odds with overall athlete skill development due to coaches focusing on their “star” athletes. This could lead to those who are perceived as less skilled not receiving feedback that could help them improve. Additionally, coaches who create an EI climate promote rivalries between teammates, and this could make it less likely athletes will encourage or help their peers to improve their sport skills.

The balance between demand and resource evaluations provide some insight into the ability of the participants to manage performance stress during the free throw clinic, which can facilitate skill development. Participants who took part in the EI free throw shooting clinic reported greater demand evaluations; however, there were no differences in resource evaluations and both groups reported a challenge appraisal. This is worth contemplating as both groups were asked to perform the same skills, received the same cues, and the only difference between the groups was the type of motivational climate that was created. The non-significant difference in resource evaluations between CTI and EI climate group may be attributed to the fact that they were all former basketball players, the expectations for the clinic were to work on developing a single, closed skill they were quite familiar with, and they knew they were participating in a brief experimental study. It is expected that in a dynamic, less controlled context (e.g., a basketball game), a CTI climate would lead to greater resource evaluations given the focus on controllable performance elements such as the amount of effort put forth and skill improvement, in addition to the positive social support received as a result of a caring climate.

While the participants in the EI climate group in the current investigation evaluated the free-throw clinic as a challenge, rather than a threat, their pattern of responses were not nearly as favorable as the CTI climate group. The greater demand appraisals and negative affect reported by the EI climate group suggest they were more adversely impacted by uncontrollable performance expectations and likely the instructors’ reactions to mistakes, despite engaging in the exact same types of activities as the CTI climate group. When also considering this in the context of a closed skill, it is interesting that participants reported greater uncertainty, stress, and perceived threats during the clinic (i.e., greater demands). It seems logical to predict that in a game where there is much more uncertainty and unpredictability that demand appraisals would be greater than in a controlled experimental setting aimed at developing a closed skill.

While the overwhelming evidence suggests a challenge appraisal will facilitate performance to a greater degree than a threat appraisal (Hase et al., 2019), the relationship is nuanced. Blascovich et al. (2003) noted that the balance between demand and resource evaluations are just one factor to consider when seeking to understand the human response to stress during motivated performance tasks. Future research should consider also examining other important indicators of challenge and threat (e.g., cardiovascular reactivity), as well as performance. To adequately measure the challenge and threat appraisal of athletes, the combination of physiological responses (e.g., heart rate variability) need to be paired with psychological variables (e.g., resilience).

This study had strengths related to its experimental and cross-disciplinary approach, as well as several limitations. Previous experimental climate research studies (Hogue, 2024; Hogue et al., 2013, 2017, 2021; Solmon, 1996), have typically used juggling as the physical activity, and recruited novice participants. To further test achievement goal perspective theory, there is a need for more sport-specific interventions to move beyond recreational activities to examine the CTI and EI climates, respectively, in more traditional sports such as basketball, and to include participants who have experience with the sport. Another strength is that the instructors and confederates were consistent across climate conditions (i.e., to avoid unique coach characteristics), and they received extensive training to prepare them to create the respective climate conditions. They met weekly across a semester to discuss climate research, practice the protocol for the clinic, and receive feedback on their performances. Interventions like this are a major undertaking and are dependent upon the skill and proficiency of the coaches and confederates to create the distinct environments.

In terms of limitations, while this study was sport specific, the free-throw clinic was brief and conducted with individuals who were not currently competitive basketball athletes. The coaches dressed like coaches, and there was a sport-like atmosphere with the clinic, but it was clearly apparent to participants that they were participating in a staged research study. There were multiple cameras on the gym floor, as these are needed to capture the biomechanical data, and this may have affected some participants more than others. Some may have felt more nervous taking shots than others, but the court conditions were the same for all the athletes. Also, only men were included in the current investigation. As a result, women athletes need to be included in future investigations in order to understand if there are relevant gender differences.

In summary, the findings demonstrate that a CTI motivational climate leads to more favorable changes in free-throw shooting biomechanics and psychological responses compared to an EI climate. Specifically, athletes in the CTI climate group exhibited movement patterns more closely aligned with proficient shooters and reported greater positive affect, effort, and motivation to continue practicing. In contrast, the EI climate group perceived the clinic as more demanding and experienced increased negative affect. These results underscore the value of integrating motivational climate strategies, particularly those emphasizing care and task involvement, into skill development settings. Future research should extend these findings by exploring a wider array of sport-specific skills (e.g., softball, track and field, tennis), using intact teams, and implementing longer interventions. Moreover, there is a need to apply such interventions within actual sport programs and to collaborate with coaches in developing a practical “CTI climate toolbox” that promotes consistent application across teams. These efforts will contribute to a deeper understanding of how motivational climate training can be implemented by coaches, administrators, and sport support staff to enhance both athlete performance and well-being.

## 5 Conclusion

The findings of the present study provide compelling objective evidence that a CTI climate enhances both biomechanical and motivational outcomes in a sport-specific context. Athletes exposed to the CTI climate demonstrated shooting kinematics that more closely resembled those of proficient basketball shooters, as defined by previously published research. Additionally, these participants reported significantly greater effort, excitement, and interest in continuing to practice the skills introduced during the clinic. These outcomes support the growing body of literature emphasizing the benefits of CTI climates over EI climates for optimizing athlete development. Importantly, this study contributes experimental and objective performance-based data to a field often dominated by self-reported psychological measures. The results can be used by practitioners and educators to promote the creation of more empowering and supportive sport environments when working with coaches, parents, and athletes. Future interventions should focus on training coaches to implement CTI principles within their teams. Such efforts have the potential to enhance athletes’ technical skill development, increase sustained effort, reduce performance-related stress, and foster long-term motivation, ultimately improving the sport experience across age groups and levels of ability.

## Data Availability

The original contributions presented in the study are included in the article/supplementary material, further inquiries can be directed to the corresponding author/s.
